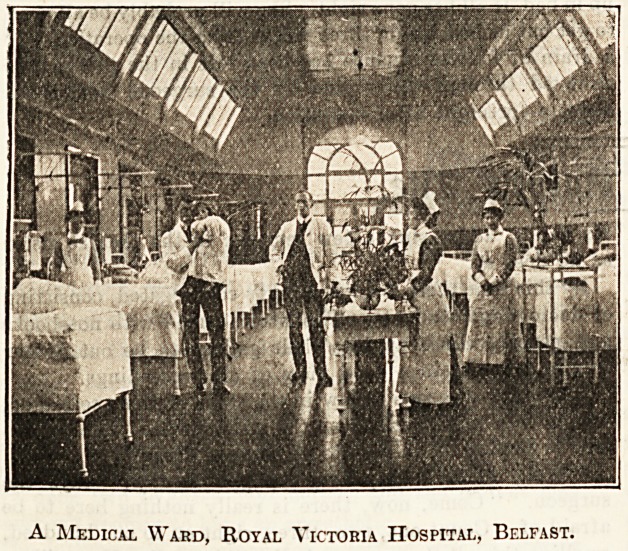# "The Hospital" Nursing Section

**Published:** 1906-09-01

**Authors:** 


### The Hospital.

### Nursing Section.

Contributions for "THE HOSPITAL," should be addressed to the EDITOR, "THE HOSPITAL" NURSING SECTION, 28 & 29 Southampton Street, Strand, London, W.C.

No. 1,041.—VOL. XL. SATURDAY, SEPTEMBER 1, 1906.

### Notes on News from the Nursing World.

### OUR CLOTHING DISTRIBUTION.

THE summer holiday season is fast waning and the majority of nurses have returned, or are about to return, to their work, all the better, we hope, for a pleasant and health-giving vacation. We shall be forgiven for reminding our readers thus early of our Clothing Distribution at Christmas; the more freely because last year some thought that we were rather late in making the preliminary announcement. The inevitable conclusion is that the longer the notice given the better is the chance of securing a record number of garments for the benefit of patients in hospitals and infirmaries. Therefore, we now appeal again for the help which is so freely afforded us in a cause that needs no recommendation. We particularly ask that no one will hesitate to send us the smallest contribution. The large parcels are greatly valued because they do so much to enable us to comply with requests from institutions urgently in need of warm clothes for the inmates, but if only 5 per cent. of our readers would send us one garment each we should have a splendid quantity to give away at Christmas. All parcels must be addressed to the Editor, 28 and 29 Southampton Street, Strand, London, W.C., with "Clothing Distribution" marked on the outside.

### IN DEFENCE OF OUTDOOR UNIFORM.

CONSIDERABLE indignation has been excited amongst Australian nurses by the wholesale condemnation bestowed on the wearing of outdoor uniform by an American monthly periodical, and a spirited attempt has been made to turn the tables. It is urged in general that the advantages of outdoor uniforms for private nurses are numerous, and in particular that it is far more sanitary, as well as far more workmanlike, than the frills, feathers, and jewellery which some American nurses affect when they go to their cases. One advocate of outdoor uniform says that her usual experience is that when she reaches the patient's house she has to begin work at once, often not having time to even put on her cap. We certainly think that there is no reason why the use of outdoor uniform should be treated as either wanting in taste or in any way opposed to the worthiest traditions of the nursing profession.

### GUARDIANS AND GRIEVANCES.

FOR some time past the Solihull Guardians have experienced considerable difficulty in retaining the services of their nurses, and, in order to ascertain the cause, a special committee was recently appointed. At their last meeting the report of the Committee was presented, from which it appears that there were complaints that necessary medical appliances could not be obtained owing to the scanty supply in the Infirmary, that the clothing for the inmates was insufficient, that the dietary for the nurses was too rigid, that they had too little off-duty, and that their laundry allowance was inadequate. In effect, it was decided that all these complaints were justified, and proposals for remedying a state of things which was clearly indefensible were adopted. It is estimated that to carry out the recommendations embodied in the report an extra expenditure of £70 a year will be involved; but we agree with the Solihull Guardians that the additional sum will not be wasted. In any case, the removal of legitimate grievances by Guardians is essential if they desire to possess a competent nursing staff.

### THE FUTURE HOME OF THE ROYAL NATIONAL PENSION FUND.

THE Pension Fund nurses do not know that the headquarters of the Fund are to be moved to a new building at 14 Buckingham Street, Strand, a large corner house overlooking the County Council's Embankment Gardens and the Thames. The old house now standing is of great historical interest. It is said that Peter the Great took up his abode here when studying shipbuilding at Deptford, and that Charles Dickens either stayed there at one time or visited a friend there, for he has described some of the apartments in "David Copperfield"; and these apartments can still be identified. There are other traditions concerning the house, but they have not been quite so authenticated. The future offices of the Pension Fund are in an excellent and convenient position.

### MORE TROUBLE AT WAIHI HOSPITAL.

A FEW weeks ago we mentioned the trouble at the Waihi Hospital, New Zealand, caused by the intervention of the trustees in a matter of staff discipline, which resulted in the resignation of the matron and two charge nurses. These resignations were withdrawn in consequence of assurances of confidence from the trustees, but there has since been further friction, with the result that the resignations have been handed in again and accepted. Subsequent to their acceptance a dispute arose in one of the wards at eleven o'clock at night between two charge nurses. It waxed so fierce that the matron, two members of the medical staff, and two of the trustees were sent for. The quarrel, however, proceeded, and the practical outcome was that the matron and one of the charge nurses quitted the institution the next morning with their belongings. Eventually the other charge nurse was installed as temporary matron, but several of the trustees are extremely dissatisfied with the position and trace all the mischief to the reinstatement by their colleagues of the nurse who was suspended by the former matron for being asleep on duty. For the sake of the patients in Waihi Hospital, we hope that whoever succeeds Miss Brooke-Smith as matron will insist upon her authority being clearly defined from the outset.

### FULL VALUE FOR MONEY.

The Sculcoates Guardians have increased their subscription to the Hull Jubilee Nursing Association from £50 to £100 per annum. Exception was taken to the proposal on the ground that, although the country parishes have to contribute to the common fund from which the subscription is made, they receive no benefit from it. But only two of the Guardians supported this view, the majority being of opinion that if the outside poor were not attended by the Queen's nurses the cost to them would be greater than it is under existing conditions. The Chairman said that "it had been proved up to the hilt that the Guardians got full value for their money." Here is another proof of the fact that Queen's nurses have become important auxiliaries of the Poor-law authorities, and there are many indications that in the future they will play a yet more prominent part in connection with the system of outdoor relief.

### ANOTHER PHASE OF THE HOLIDAY QUESTION.

At the last meeting of the Birkenhead Guardians a recommendation of the House Committee to allow the superintendent nurse leave of absence for fourteen days was met by the objection that holidays ought not to be granted to officers with less service than a year. As the superintendent nurse was appointed early in 1906, this objection was obviously aimed at her, and we are glad that a majority of the Guardians refused to adopt the view that she is not entiled to a summer holiday because she has not been in their service for twelve months. It is a satisfactory feature of the discussion that one of the speakers strongly supported the recommendation of the Committee "in the interest both of efficiency and of common humanity."

### ONE NIGHT NURSE FOR TWO BLOCKS.

At the last meeting of the King's Lynn Guardians the Rev. A. H. Hayes stated that, in consequence of the discharge of one of the nurses, there was now only a single nurse to look after the two blocks of buildings at night, and that the medical officer did not consider this to be sufficient for the adequate care of the patients. In spite of the fact that the question had been brought before the Guardians on a previous occasion, the Chairman again urged delay, and nothing was done. If anything untoward happens to the patients while there is but one nurse on duty during the night in two blocks of buildings, the blame will justly be attributed to the Guardians, who have no right to impose an excessive burden upon an individual member of the staff.

### CRICKET AND DISTRICT NURSING.

We congratulate that splendid cricketer, Mr. Thomas Hayward, upon his choice of the charity to which the receipts of the annual contest arranged by him at Cambridge to take place at the end of the cricket season will be devoted. The popular professional desires that the District Nurses' Fund in the University town shall have the benefit of the gate-money at the match, which will be played on the 22nd between his eleven and eighteen local cricketers. The eleven, it is hoped, will include Lord Dalmeny, Mr. Knox, and Mr. Crawford, each of whom has done so much this year to advance the reputation of the Surrey team. The fixture is sure to be a great attraction, and we trust that fine weather will help to render the sum available for the cause as substantial as Mr. Hayward himself could wish.

### NURSES' SOCIAL UNION.

Summer has not interfered with the growth of the Nurses' Social Union, which has now sent its branches all over Somersetshire. Four delightful meetings have been held lately. At Bath the gathering took the form of a garden-party, and between forty and fifty nurses availed themselves of Mrs. T. B. Silcock's invitation, and spent a most enjoyable afternoon in the midst of flowers and fruit. The Minehead and Bridgwater branches have been equally fortunate, as, by the kindness of Mrs. Sterry, of Chapel Cleeve, and the Hon. Mrs. Stanley, of Quantock Lodge, the nurses of those branches spent a very pleasant afternoon amidst beautiful surroundings which they thoroughly appreciated. At each of those meetings a lecture on "Nursing Expedients" was given by Mrs. Lance, who has herself been a nurse, and was welcomed as an old friend by many of those present. Special interest attaches to the first meeting of the Yeovil Branch, which was held by the invitation of the local organiser, Mrs. Fowke, at her house. The objects of the Union were first explained, and this was followed by a suggestive and interesting lecture by Dr. Penrose Williams on some of the latest developments of medical science. Then came tea in the garden, charmingly arranged on little tables, so that the nurses could form their own little parties. As the work of the Union develops and strengthens, many instances arise of its usefulness. Fresh friendships are formed and old ones revived, and as many of the nurses in Somerset have been at the same training school it provides opportunities for the meeting of old comrades which are heartily welcomed.

### ST. KATHERINE'S HOSPITAL.

The Rev. Canon Holmes has been appointed to become a member of the Chapter, and has not been appointed Master, as reported. The Rev. A. Peile is Master of St. Katherine's Hospital.

### SHORT ITEMS.

The Chelsea Hospital for Women has named a bed the Hannah Finnie bed as a memorial of the late Mrs. Hannah Finnie and her generous bequest.

### The Nursing Outlook.

"From magnanimity, all fears above;
From nobler recompense, above applause,
Which owes to man's short outlook all its charm."

### NURSING IN THE UNITED STATES.

The radical section of nursing opinion in this country had its origin in the example set by certain advanced spirits in the United States of America, who may practically be held responsible for the ideas and claims which the British section of advanced opinion have set forth with so much energy and declamation. It may be interesting, therefore, to draw attention to the circumstance that, in fact, the United States had few or no schools for the training of nurses until quite recent years. The returns of the United States census for 1904 contain the names of 724 schools of nursing, of which not more than 100 were in existence in 1890, including both hospitals and asylums. If we eliminate the asylums we find that there were considerably less than 100 schools of nursing in the United States fifteen years ago. These figures eloquently testify to the need for caution in accepting opinions which rest upon so relatively limited an experience.

Our American cousins enjoy a climate so stimulating that it acts like champagne upon the nerves and energies of the people, who in consequence wear themselves out by hustling always in all things, to the injury of their own health and the hindering of steady progress on lines resting upon matured and deliberate judgment. The charm of the Americans is no doubt largely due to the quaint boldness and originality which often characterises the point of view which they express with hasty volubility. There can be no doubt that immense progress has been made by the exhibition of this hustling spirit, but it has been attained at the cost of the individual worker, and it is a sad fact that in the United States a host of able minds are practically worn out at an age so young as to produce nervous breakdown of a permanent character before middle life has been attained. It is not surprising in such circumstances to find that nursing has been affected materially in the United States by the circumstances under which individuals carry on their work in that country. Dr. Phelps, in a recent paper in the *Trained Nurse*, has shown that there are only eight or ten nurse-training schools connected with hospitals of 500 beds or upwards. There are about 175 such schools in connection with hospitals of from 100 to 500 beds, and 475 other schools connected with hospitals having less than 100 beds.

One strange feature connected with nursing in the United States is the correspondence schools, which, as Dr. Phelps points out, advertise in high-sounding language, and hardly try to conceal the fact that they are working for the fees. Some of these schools may give a diploma in six months to a person whom they have never seen, and to one who may never have nursed at all. It is not to be wondered at that a movement is now on foot to prevent such so-called graduates from using the title of trained nurse.

Nurse registration has made some progress in the United States of America, seeing that nine of the forty-five States which constitute the Union have passed registration laws. Thes laws enforce practically one common rule—*i.e.* that the applicant should be a graduate of some recognised school, have been engaged at least two years in studying the practice of nursing, and shall in addition pass an examination and pay a fee. None of these laws prevent anyone from doing nursing work; they only prohibit the use of the name trained or registered nurse. One effect of these laws has been to exhibit the impracticability to control the majority of the so-called nurse-training schools which are attached to the smaller institutions.

Another effect of registration has been to increase the fees which nurses are able to obtain, and the tendency seems to be in an upward direction. The result is that none but the very rich can afford to pay for a trained nurse in the United States, and that the majority of the people whom we denominate the middle classes are left without skilled nursing in the hour of sickness. The relatively poor, or those who have so little self-respect as to take free medical relief wherever they can obtain it, have, of course, the advantage of trained nursing, but the great bulk of the people find themselves in a difficulty when they or members of their family are attacked by illness. This is no doubt one reason why nursing homes and paying wards of hospitals are to be found in most cities of the United States. The exaction of high fees is tending to alter the opinion of the public in regard to nurses. Trained nurses are coming to be regarded more and more as luxuries, and many of the higher and more attractive qualities which have won world-wide esteem for the nursing profession are neither expected nor forthcoming. Another feature of American nursing of the present day is a recent effort to supply cheaper attendance in the day of sickness by training women as domestic nurses through classes of instruction which provide in a few weeks or months sufficient study and practice, as it is claimed, to meet the more ordinary needs of private patients in middle-class families. Altogether the position of nursing in the United States is in a state of transition due largely to the rapidity with which existing methods have been developed in recent years.

### The Care and Nursing of the Insane.

By PERCY J. BAILY, M.B., C.M.Edin., Medical Superintendent of Hanwell Asylum.

### I.—ANATOMY AND PHYSIOLOGY.

(*Continued from page* 286.)

*The Brain.*—When one looks at the human brain (fig. 19) it is easy to see that it may be roughly divided into three chief parts which differ obviously

FIG. 19.—THE HUMAN BRAIN.
*a*, cerebrum; *b*, cerebellum; *c*, medulla oblongata; *d*, spinal cord.

from one another in their shape and size and in the nature of their surfaces. These three parts are called—

1. The large brain or cerebrum.
2. The small brain or cerebellum.
3. The pons and medulla.

The total weight of the brain averages about, or a little over, three pounds, and is about five ounces less in women than in men.

1. The large brain or cerebrum, as its name implies, is by far the largest of these three divisions. It occupies the greater part of the skull cavity, and extends from behind the forehead backwards over the hind brain or cerebellum, which it overlies and which, when looked at from above, it almost entirely hides, and its general outline corresponds with the vaulted interior of the cranial cavity. Running along its surface from before backwards there is a deep central fissure which dips deeply down into its substance, and divides it into two equal parts which are called the right and the left hemispheres. The surface of the hemispheres presents a very characteristic appearance, being composed of a number of irregularly arranged elongated, almost worm-like elevations which are separated from one another by depressions (fissures or sulci) into which dip prolongations of the pia mater. These elevations, which seem to twist and interlace with each other, are called the convolutions of the cerebrum, and their meaning is to give a greater extent of surface to the brain, which, as we shall presently see, is composed of grey matter. The greater the number of convolutions and the more intricate and apparently confused their arrangement the higher is the intelligence of the individual possessing them. Hence we find in the lower animals, such as fishes and birds, the cerebral surface is much smoother and the convolutions more simply arranged than in the higher animals, such as the apes and man and in the lower orders of man, such as the Hottentots and other savages, than in the white races. The convolutions are developed by education and training, and so increase in complexity with the growth and advancement from childhood to maturity, and they undergo some shrinkage as old age creeps on and brings with it the natural enfeeblement and simplicity of our second childhood. The two cerebral hemispheres are connected with one another by a broad, flat band of nerve fibres, which pass from one side to the other. This band of fibres (called the corpus cullosum) lies at the bottom of the deep central fissure which divides the two hemispheres from one another. The internal arrangements of the cerebral hemispheres are somewhat complicated, and need not now occupy our attention.

2. The small brain or cerebellum is relatively to the cerebrum very much smaller in man than in the lower animals. It occupies the lower and hind portion of the skull cavity, and is, as we have already seen, quite overlapped by the posterior portion of the cerebrum. It, like the cerebrum, is composed of two equal and similar halves, but the depression which separates these from one another is comparatively shallow. Its surface is masked by numerous closely-set fissures which run more or less parallel to one another, giving this portion of the brain the appearance of being made up of a series of closely-packed layers.

3. *Pons and Medulla.*—The bridge (pons) is a bunch of nerve fibres which pass from side to side. It looks something like a small cushion. The bulb (medulla) is really the upper enlarged end of the spinal cord. It consists largely of nerve fibres on their way from the brain to the spinal cord, and *vice versa*. The intermediate brain forms a sort of junction whereby the cerebrum and cerebellum communicate with each other, and both these with the spinal cord.

The spinal cord is an elongated rope-like mass of nervous tissue which is a continuation of the bulb. There is no definite alteration in the structure of these two parts which indicates where the one ends and the other begins; as soon as it passes out of the skull into the spinal canal the mass of nerve tissue ceases to belong to the bulb, and becomes the spinal cord. In the middle line, both in front and behind, the surface of the cord is indented by a fissure; both of these fissures pass towards the centre of the cord, but do not meet, but they almost cut it into two equal halves. Running through the centre of the cord there is a small canal which contains fluid (cerebro-spinal fluid), and which communicates with a series of cavities (the ventricles) in the bulb and great brain. From the sides of the cord there branch off from it the series of spinal nerves which pass out from the spinal canal between the vertebræ. Of these there are 31 pairs.

### THE CARE AND NURSING OF THE INSANE—*continued.*

Each spinal nerve arises from the cord by two roots (fig. 20), as they are called; these almost imme-

Fig. 20.—Section of Spinal Cord, with Roots of Spinal Nerves—Front View.

1, 1, sensory root; 2, 2, combined nerve trunk; 3, 3, motor root. (N.B.—No enlargement or ganglion should appear on the anterior root, only on the posterior.)

diately unite with each other, and form what is called a mixed nerve. The meaning of this term we shall presently understand when we come to inquire into the functions of these parts of the nervous system.

Both the cranial and the spinal nerves are, generally speaking, mixed nerves. They are composed of a large number of nerve fibres, just as a skein of worsted is composed of a large number of individual threads. After leaving the skull and spinal column the individual fibres of which they are composed separate from one another, forming branches of the mixed nerves which pass to all parts and to all the tissues and organs of the body.

*The Arrangement of the Grey and White Matter.*

1. *In the Great Brain.*—The whole of the surface of the great brain is covered with a layer of grey matter, over which it spreads in a continuous sheet which is about ¼ in. thick. The object of the convolutions of the brain is to allow of an increase in the extent of this sheet of grey matter, and it follows that the more numerous and more complicated these folds and puckers are the greater will be the amount of grey matter. In addition to this extensive sheet on the surface of the great brain there are many patches of grey matter scattered deeper in the substance of the cerebrum. These secondary patches (basal ganglia, as they are called) are connected by nerve fibres with the superficial area, and are under its control. The other parts of the great brain are composed of white matter.

2. *In the Small Brain* the grey and the white matter have the same relative position to one another, as in the great brain—that is to say, the surface is composed of grey matter, while beneath this is the white matter, amongst which are found again some secondary patches of grey matter.

3. *In the Intermediate Brain* and *in the Spinal Cord* there is no grey matter on the surface, which is composed entirely of white matter. This in the spinal cord encloses the more deeply situated grey matter almost as in a tube. In the bulb and bridge, where the central canal of the cord widens out to form ventricles, the grey matter is arranged around these.

Throughout the whole nervous system the grey matter is much more abundantly supplied with blood than the white. This fact indicates that the grey matter requires more nourishment than does the white, and that the nutritive or chemical changes, whatever these may be, which are inseparable from vital activity, take place rather in the grey than in the white matter.

(*To be continued.*)

### The Nurses' Clinic.

### ABDOMINAL HYSTERECTOMY CASES.

The operation of hysterectomy is done by the surgeon when necessary, with the consent of the patient, in cases of disease of the uterus, either malignant or otherwise. He may perform the operation either vaginally or abdominally, the latter being the most usual way. If the disease be malignant and if the patient have much or foul discharge the surgeon sometimes finds it necessary to curette the uterus preparatory to the operation. The curette is generally done two to three days before, and the patient may or may not require to have an anæsthetic.

The instruments necessary for curetting will be uterine dilators, curettes, duck-bill speculum, douche nozzle, Bozemann's catheter, uterine sound, vulsellum forceps, scissors and dissecting forceps, Playfair's probe (topped with cotton-wool), large strips of iodoform gauze, sterilised water in a small vessel for the surgeon to clean curette and save scrapings from uterus, irrigator filled with antiseptic solution, pad of gamgee, T bandage, and pure carbolic, which may be used on Playfair's probe for swabbing out the uterus after operation. The surgeon usually packs the uterus and vagina with a plug of iodoform gauze, which he orders the nurse to remove on the following day, after which the patient is douched twice daily until the abdominal operation.

The preparation of the patient for operation will be as follows: An aperient must be given on the afternoon previous to the day of operation. The whole of the abdominal surface must be shaved; the patient must have a bath, after which the skin of the abdomen is thoroughly washed with soap and warm water, then cleansed with turpentine, methylated spirit or ether being used to clean up. The part must then be washed with antiseptic solution, perchloride of mercury 1-1000, carbolic lotion 1-40, or any other solution preferred by the surgeon; a compress of sterilised lint being wrung out of the same solution is laid on the part prepared and covered with a pad of gamgee. This should be firmly bandaged on with a double spica, and the patient must remain in bed, in case of disturbing the dressings.

During the process of preparation the nurse must have her hands surgically clean, and must use sterilised swabs in each case of cleaning the surface to be operated on. In the early morning the patient's bowels must be thoroughly cleared out by a large soap and water enema. Breakfast of tea and toast may be given four to five hours previous to operation.

The patient's bladder must be emptied by a catheter immediately before she goes to the operating theatre. Her chest, back, and front must be protected from cold by a gamgee jacket, and she may wear a short flannel jacket coming to the waist, fastened behind, and long stockings. When the patient is on the operating table a blanket is spread over the lower part of body and another over the chest. Such cases are usually operated on in Trendelenberg's position.

The instruments etc., necessary will be scalpel, 12 pair of artery forceps, two pair of dissecting forceps, scissors (angular, straight, curved on the flat and long uterine), pedicle clamp forceps (straight and curved), vulsellum forceps, ovum forceps, myoma forceps, blunt pedicle or hernia needle, retracters; curved, half curved, and straight cutting needles (large and small); intestinal needles; needle-holding forceps; silk ligatures (stout, medium, and fine); catgut and silkworm gut sutures; swabs of sterilised gauze; and long abdominal wipes with tapes attached; dressings of sterilised gauze and pads of sterilised gamgee; and many-tailed or ordinary bandage. Large strips of iodoform gauze may be required for packing purposes. Sterilised towels, large abdominal towel with a slit in the centre, a good supply of sterilised water and antiseptic solution will also be necessary.

The following things ought to be ready in case of emergency: transfusion apparatus, with aneurism needle; saline solution, ʒj. to the pint; hypodermic syringe with strychnine must be kept at hand as in all other operations where an anæsthetic is given. Before the surgeon closes the abdomen it is necessary that the nurse in charge should count the abdominal wipes, to make sure that she has the same number as when the operation began; she must also see that she has the correct number of small forceps, which the surgeon will ask her to count. When the operation is over, the patient must be kept warm in bed with hot-water bottles and blankets, a cradle being used to lessen the weight of the bedclothes, and a pillow to support the knees. If retching or vomiting occur the nurse must place her hand firmly on the abdomen each time, so as to save rupture or bursting of the stitches. For the first twenty-four hours very little, if anything, should be given to drink. The mouth may be washed occasionally with water if the thirst is very great, and in this case sometimes a pint of tepid water may be ordered to be given by rectum, which often alleviates the thirst. If after the first twenty-four hours the chloroform-sickness has passed off, the patient may be allowed to have fluids in small quantities by mouth, beginning with sips of water, afterwards milk diluted with kali or plain water, tea, chicken soup, beef tea, and barley water may be given ℥iij. to ℥iv. hourly. An aperient is generally given about the third night after operation; when the bowels have acted well, solid diet may be started, beginning with a little toast or thin bread and butter, or milk pudding. The next day a little fish may be given, and so on until the patient is gradually got on to ordinary light diet.

This operation being a severe one, with a great amount of shock to the system, the patient will require to be carefully watched for the first few days and all symptoms reported to the doctor in charge. A four-hourly (temperature, pulse, and respiration) chart ought to be kept for the first week. The urine must be measured and charted daily for a week. The patient should be encouraged to pass urine herself from the first, but in case of her not being able to do so, the catheter will be necessary, but not oftener than eight-hourly; surgical cleanliness being observed by the nurse each time. If the patient complains of pain and flatulence she may be relieved by the use of the rectal tube. For the first day or two after operation there may be severe collapse, in this case the doctor may order enemata of saline and brandy or beef tea; and the foot of the bed may be raised with blocks. Sometimes, when the shock is very great and the patient has lost a large quantity of blood, the doctor may find it necessary to transfuse saline solution into the veins.

After the first five days the patient is fairly well out of danger. The stitches are generally removed by the surgeon about the end of a fortnight, and the patient allowed to sit up in bed, if all has gone right. She will probably be allowed to get out of bed inside three weeks from the operation. If a drain of iodoform gauze has been left in the pelvis it is removed through the vagina by the surgeon; sometimes about a fortnight after operation.

### Incidents in a Nurse's Life.

### OPERATING DAY IN A NEW ZEALAND HOSPITAL.

WE have been busy all the morning preparing the theatre for its work. From the large windows there is a fine view of the bay. The cool water seems very tempting on a hot, dusty day when we glance up during the operations, with a very small chance of getting out for a row or sail. Many is the disappointment we have had when it has been "our afternoon off," and we have made all arrangements to go out in the boat. It is a very usual occurrence to be kept late in the theatre at an operation, and a glance through the windows shows us the boat merrily gliding down the bay—without us! But duty first. In the corner of the theatre stands the steriliser, bubbling away whilst it is destroying the numerous microbes that lurk amongst the dressings and sponges, which are rapidly becoming "cooked" within its spacious bosom. Everything is spotlessly clean.

In the instrument-case lies row upon row of brightly-polished instruments, which carry life and healing on their sharp edges to many an unconscious victim. Dressings, sponges, towels, lotions, and instruments now are all out in readiness for the surgeons.

A quick step on the stair, and one surgeon enters. Divesting himself of his coat, he takes a sterilised overall which the nurse hands to him, and slips it on. Then, giving a comprehensive glance round the room, he turns to perform that most necessary and elaborate ceremony, that of washing his hands, addressing a few remarks to the theatre sister as he does so.

By this time there is quite a party congregated, consisting of doctors and students. The latter, armed with notebooks and pencils, climb up into their gallery to be out of our way and to obtain a good view of all proceedings.

The other surgeons follow in the steps of their colleague, who now is busy overlooking the instruments. The patient now is coming in, in a state of nervous dread.

"Well, Mrs. Jones, how are you to-day?" says the surgeon. "Come, now, there is really nothing here to be afraid of. Count one, two, three, right on to one hundred, smelling this all the time, and all will be well. You will go off to sleep, and when you wake up all will be over, and you will be back again in your own ward." The quiet, reassuring tones of the surgeon give her confidence, and after a few minutes the counting becomes confused. She feels as if she were being carried up, up, and then all is oblivion.

Then sister loosens her hands, which all the time have been firmly clasped by the patient, and removes the bandages and dressing. The surgeons commence their work quietly and earnestly, without an unnecessary word being spoken. The surgeons and nurses, alike clad in their long white overalls, white caps, with arms bare and scrupulously clean, look very like priests and priestesses engaged in a very solemn ceremony.

INCIDENTS IN A NURSE'S LIFE—*continued.*

The silence is broken by the operator pausing in his work. "Is she all right?" he asks. On receiving the affirmative, works away as before. Nurses fly here and there. Before the doctors or sister has time to ask for anything, willing probationers are there with the required lotion, each trying to be more smart than her fellow "pros."

Sister is looking very worried. She is counting her sponges most carefully as one by one they disappear into the capacious wound. Then, as the correct number are once more returned to her, she gives a sigh of contentment.

A few sutures—"Silk, please!" and then as the surgeon glances up at the theatre clock, ticking away—the only sound heard in the theatre,

"Thirty-five minutes," he says, with his hand on the patient's pulse.

"Give one-thirtieth Strych.—hypodermically, if needed; and she may have one-sixth morphia if in much pain." Then, "Next, please!"

Very quickly Mrs. Jones is removed from the table to a barouche, and wheeled into her ward, where bed and hot bottles, cradle, etc., are all in readiness.

The "pros." are busy cleaning up the theatre for the next victim, and the surgeons are enjoying a cigar or cup of tea for their fifteen minutes' interval.

And so the day goes on until dusk. Then, as the last case leaves, everyone gives a sigh of relief.

There is a great "cleaning up" after the surgeons and students all have departed. The nurses work on, replenishing lotion-jars, cleaning instruments, etc., tired out, but chatting over the cases between whiles. They must leave everything ready for an emergency—every bowl, dish, mackintosh, and instrument must be left scrupulously clean, for who can tell when the theatre will be required next? No one.

At last everything is done. Windows are all opened; the cool evening air is blowing gently in, gradually freshening up the heated room, and "operating day" has once more come to a close.

### Visit to a Famous Irish Hospital.

The Royal Victoria Hospital at Belfast is very pleasantly situated on a leading thoroughfare within a short distance of the centre of the city, facing a pretty little park, gay in summer with flowers and sparkling fountains. It is equally near the open country, and almost under the wing of a heather-crowned mountain where many delightful "evenings off" have been spent by members of the nursing staff. The entrance to the grounds is formed by an arch resting on the porter's lodge on one side, and the bicycle shed on the other, from which a wide carriage drive sweeps up to the front porch, the latter being surmounted by a handsome statue of Queen Victoria. The hospital is of red brick, ornamented with stone facings—it consists of a long low main building, having two five-storied wings built one on either side of the front porch. These quite shut off the view of the main body of the hospital from the front. The porch opens into a large square hall, whose floor and walls are entirely composed of Italian marble, the stained-glass ceiling representing the signs of the Zodiac. Opening directly from the hall opposite the porch entrance runs the main corridor from east to west, some 450 feet long and 9 feet wide. Branching southwards are the wards. They are built side by side, being lighted from the roof with lantern windows, and at the south end of each, extending its whole width, is a balcony with French casements overlooking the tennis courts and croquet ground, from which a good view of the asylum grounds which adjoin, and of the neighbouring hills, is obtained.

A Medical Ward, Royal Victoria Hospital, Belfast.

### In the Wards.

The wards are divided into "units," consisting in the medical or east end of one large and one small male, and one large and one small female, ward. They contain respectively fourteen beds in the large and two beds in the small wards, a ward kitchen, a clinical room, a class-room, two lavatories, and two bath-rooms. In the surgical or west end of the building is the same accommodation, with the exception of clinical and class-rooms. Instead of these, they have an operating theatre. The "units" are built with short passages branching from the main corridor with clinical and class-rooms in the centre, the kitchens being so planned that the nurse can see and hear all that goes on in both male and female wards, while she makes the tea, etc. The brightly-polished floors, the white paint of the furniture, and the "brights" and glass of the lockers all combine to give an impression of freshness and snowy cleanliness not always met with even in the wards of an up-to-date hospital. Altogether, there are three hundred beds, but unfortunately two units containing thirty-two beds each are at present closed owing to want of funds.

### The Plenum System.

The hospital was especially designed and built for the Plenum system of heating and ventilating. This is carried out as follows: Large fans, driven by steam, draw air from the outside through wet screens, which filter it from all soot and dust. It then passes over rows of steam pipes which warm it, and it is driven by the fans into a large tunnel running underneath the main corridor, from whence there branch off smaller tunnels with openings into each ward. There are also counter-openings in the wards for the escape of foul air, which passes into separate outlets. There is thus a constant influx of pure, filtered air to every part of the hospital. The prevailing atmosphere is very agreeable, the place being comfortably warmed in winter and refreshingly cooled in summer. The temperature never varies more than a degree, and it is really wonderful how well "cases," both medical and surgical do, especially "chests." The absence of flies, those troublesome pests, has been frequently commented upon by both visitors and residents, and the phrase, "There are no flies in the new hospital," has become quite a saying with the students of the local medical school.

### The Absence of Stairs.

A special feature is the absence of stairs, all the wards being on the same level. The extern, a spacious hall of quite ecclesiastical appearance with its coloured-glass windows, is of no little importance considering that between 23,000 and 25,000 new cases pass through it annually. The floor here and also in all corridors, kitchens, bath-rooms, lavatories, and operating theatres are of terrazzo, those in the wards being polished wood. The electric light is laid on everywhere. In the grounds will be noticed two pretty villas, one is devoted to "septic" cases, the other to "infectious" ones.

### The Nursing Staff.

The west wing is entirely given up to the nursing staff. On the ground-floor are the matron's office, dining and sitting-rooms, the nurses' and sisters' dining and sitting-rooms and the parlour-maids' pantry. As far as possible, each nurse has a bedroom to herself, prettily furnished in oak, the sitting-rooms being abundantly supplied with easy-chairs, sofas and cushions, and made bright with plants. There is an excellent lending library for the nurses, under the charge of the Home Sister, books being given out weekly, and a never-failing supply of hot water in the numerous bath-rooms. The east wing provides accommodation for the resident medical staff, and for the servants of the entire building, under the charge of the housekeeper. The nursing staff consists of the matron, the assistant-matron or home sister, the night superintendent, nine day sisters, twenty-four staff-nurses, and fifty-five probationers. The period of training is for three years, probationers in their third year being promoted to staff nurse's rank, provided they have passed a qualifying examination. They receive lectures during each of their three years' probation on medicine, surgery, gynæcology, ophthalmology, x-Rays, and electricity, from the members of the visiting staff, and on cookery from the housekeeper, and an examination is held at the end of each course of lectures. Probationers pay no fee, they receive no salary the first year, £15 the second, and £20 the third. Three weeks' holiday is given every year, two and a half hours' off duty daily, and a day once a month, permission being given to go off at 6.30 P.M. the evening before to enable those who have friends in the country to have a night away from town. In addition to this, staff nurses and sisters have a "long evening" weekly from 4.30 P.M.

### The Throne Hospital.

No account of the Royal Victoria Hospital would be complete without some mention of the "Throne Hospital," which belongs to the Committee of the Royal Victoria Hospital. It is worked by a resident matron, under the direction of the authorities at the larger institution. It stands in spacious grounds beautifully situated on the side of the Cave Hill, about four miles from Belfast, commanding a fine view of Belfast Lough, and in fair weather the Scotch coast can be seen in the distance. It provides accommodation for the convalescent children and consumptives from the Royal Victoria Hospital, each class being nursed separately.

### Should there be Training Schools for Matrons?

BY A MATRON.

To one who knows the inner life and working of hospitals and similar institutions, the question must often present itself whether the previous life and preliminary training of the women at the head of them have been the best it is possible to conceive to fit them for their present responsible position? Could not the petty strife and jealousies, the bitterness and hardness, which too often mar institutional life and nurses' characters be often prevented if the matrons were more really capable as organisers?

But before determining what would be the best training for a Matron of a fair-sized institution, her duties and necessary qualifications must first be considered. Her work must be chiefly that of organisation and general supervision —arranging that the wards are adequately staffed, that the probationers by doing varied "duty" and receiving useful lectures, etc., may have as fair and good a training as the institution can afford; that the domestic arrangements of the "home" are such that when off duty the nursing staff may live in comfort, and in as near an approach to a home life as the surroundings will permit.

Then a great deal of correspondence must pass through the Matron's hands concerning patients, applicants for various posts, visitors, tradesmen, etc. With this large and varied scope for work, so centered is her habit of thought on ward duties that the complaint is often made that the Matron interferes with matters which should, in my opinion, be left to the responsibility of the nurse in charge of the wards. Granted that the charge nurses are efficiently qualified for their position, and are women of high character, the Matron's supervision of ward management seems unnecessary. On the other hand, the Matron should be so keen an observer, have so clear an insight, that in her daily round she should be able to judge from the general appearance of the patients, the cleanliness, the attitude of the probationers, and the respect commanded by the nurse in charge, whether or not the ward is efficiently staffed. It is often the Matron's duty to engage both probationers and qualified nurses, and it has been sometimes stated that she cannot do this without thoroughly understanding herself the duties required of them. Surely in the case of a trained nurse the opinion of her former doctors and matrons as to her general fitness, combined with—when the nurse is coming fresh from her training school—a summary of reports from those under whom she directly worked is an all-sufficient guide to the intelligent woman having a personal interview with the applicant; and in the case of a probationer it is customary that she comes for some time "on trial," when the sister of the ward is the one best able to judge of her fitness.

The successful Matron must be a woman of a broad and liberal education and wide experience, a skilful up-to-date organiser, a tactful manager, with a wide knowledge of human nature and its needs, and a keen sense of justice. Under existing arrangements a girl enters a hospital, sometimes with little education and small general experience; she works hard both with mind and body, studies and passes examinations, but her work is rightly occupied with the care of the sick. She then spends, perhaps, several more years as staff nurse and ward sister, when she practises what she has learnt and teaches it to others. But it is very obvious that there is little in all this work to fit her to undertake the government of an institution, with its varied departments and organisation. That the present system of promoting ward sisters with little other experience, through the posts of home sister and assistant matron to that of matron, is unsatisfactory, is found by their frequent incapacity to deal with general matters. What other professional women complain so much as nurses of inconvenience through mismanagement of holidays, etc., or who suffer so much from want of consideration in matters of the home life? Or what community of women is so unsociable? A room full of nurses will sit a whole meal through without speaking a word, for the home sister at the head of the table cannot talk of anything more interesting than "her cases" and "her training school," and so saturated is her mind with ward etiquette that a group of newcomers, with fresh ideas, naturally starting a conversation, receive such quelling looks—if they are not actually told to be quiet—that they soon become subdued to the general level of dullness. The consideration of these details, trivial perhaps, but which, nevertheless, have much to do with the making or marring of institution life, leads back to the opening question, and suggests the inquiry, would not some of the five, seven, or more years usually spent by the coming Matron in the wards be better passed in more general training, first at college and in general subjects, followed by study at a school of domestic science, and at some period a good home training in domestic duties and management?

### "In Coster Clubland."

BY A QUEEN'S SUPERINTENDENT.

Some of the happiest hours in my life have been spent "off duty" amongst the coster and flower girls in a London slum.

The "parlour" where they met during the winter evenings was simply furnished, warm, and bright. The girls at first were somewhat reserved, but became very friendly after a few meetings.

By what mad freak I consented in the first place to address them I never knew, and no sooner was the promise made than I bitterly repented, but could not withdraw. A week of sleepless nights followed (for I had never spoken in public), and then the dreaded hour arrived. I had been asked to give them a simple talk on their own personal health, and having no knowledge of that class of girls, I had not the ghost of an idea what this meant, and so carefully prepared a closely written essay which might have passed, perhaps, as one on elementary hygiene.

A terrible attack of "stage fright" came on as I entered the room and encountered the steady, scrutinising gaze of over thirty pairs of eyes. My knees shook, my heart was beating wildly, and my tongue refused to move.

Suddenly a voice from the far end of the room broke the silence:

"Crikey, Sal, blest if it h'aint a live nuss."

"So she are," was the reply, only she h'aint got a flyer on 'er bonnet."

The Sister-in-charge stopped the talking, and I began to read. In a few minutes their attention entirely ceased, and my voice was nearly drowned in the buzz of conversation. I looked up in sheer despair, a cold perspiration breaking out from every pore. The momentary pause checked the talk, and in the moment of terror every detail about the girls struck me: the pale shrewd faces, with the heavy fringes overhanging their eyes, huge hats with bright blue and red feathers piled on each, long brass earrings, coloured neck kerchiefs; and suddenly a thought came to me, "These are the coming wives and mothers, they will have children to mould and develop some day"; and a great longing to make them realise that they were women came to me, but at first no words. They were still silent. "Girls," I said, "I can't speak very well."

"Yer carn't that," was the honest rejoinder.

And then something in the faces gave me an inspiration, and words came (I never knew what I said), and so half an hour passed away and the ordeal was over.

I went round to shake hands with each of them afterwards. They were quite ready to discuss their daily work, and many of them were very quaint in their remarks.

"I wouldn't 'ave yer life for somefing," one of them volunteered. "Wy, I should f'int if I see'd a leg come orf anybody."

Another girl was sitting apart very sadly.

"Liz h'aint well," said one of them. "I h'aint," said the invalid. "Not one blessed wink 'ave I slept for a week. Blest if I knows what I am a-comin' to with this pain in my liver."

I looked at her, and felt rather surprised, but advised her to see a doctor. Curiosity got the better of me. A coster girl able to locate her liver!

"By the bye, where is your liver," I asked.

"Don't yer know that, bein' a nuss," she inquired scornfully. "'Ere, in corse," placing her hand over her right temple and eyebrow, "and," plaintively, "it do beat."

Earnestly I assured her that she had been misinformed. It was useless. "Who should know where me own liver is if I don't meself?" she asked indignantly. There was nothing more to be said, so with renewed expressions of sympathy I wished her good-night.

Thus passed the first of many happy nights, extending over two years.

### The Final Cookery Lesson.

BY AN AUSTRALIAN CORRESPONDENT.

"You will be given your examination papers after my demonstration this evening, and the practical examination will take place this day week" said the lady presiding over the State department cookery classes. All eyes were directed to the blackboard upon which was written "Mulligatawny soup; oyster scallops; shortbread;" we all knew that there was no peeping into those papers until these dainties were served. We had all been through the mill once over hospital work, but this was a horse of another colour. However, with the proverbial cheerfulness of nurses, we made the best of the business, chaffed each other between the courses, and forgot all about rocks ahead! At 8 o'clock the papers were produced; each one narrowly scanned the seven questions, six of which must be answered :—

1. State fully chief rules for cooking vegetables; name the exceptions, and give reasons.
2. What is baking powder composed of? Of what use is it? Explain its action?
3. Enumerate six primary ways of cooking meat. State fully how you would roast a loin of mutton weighing 5 lbs.
4. Give general laws for making and baking pastry. State fully how you would make flaky pastry, and what it is used for.
5. What effect has heat on meat, eggs, milk, and rice?
6. Of what does raw beef-tea consist? Give six rules to be observed in the preparation of sick-room cookery.
7. Explain the terms: Bouquet garni, croutons, sippets, kneading, simmering.

At 9.30 a suggestion was made that we could disperse, and a very subdued twelve rose to their feet and handed in twelve trembling papers. "If I get a sweet omelette and puff pastry on top of that paper I shall be done," exclaimed one poor victim. "Those were very unfair questions" said another who felt weak, "We were never instructed in six rules to be observed in sick-room cookery." "Your imagination surely could supply those." "Imagination! that all went into the baking powder." "I never remember whether its green or root vegetables that are cooked with the lid off.

I am sure to have reversed the whole thing." And in the distance a voice was heard plaintively demanding what effect has heat on milk. Seven days later——. Twelve cookery pupils, in spotless aprons, stood at their respective stoves—waiting. Not an examination paper, but a list of dishes to be prepared, cooked, and served—the finishing test of the twenty lessons. The tickets were given out and business gleamed from every eye; for two solemn hours not a sound was heard but the whisking of eggs, the stirring of pots, and the closing of oven-doors. The practical part was enthusiastically taken up and the pupils have entered for an advanced course. Probably before long many nurses will have qualified as certificated teachers in cookery and the hospital difficulty on this subject will be at an end. The results of the examination have not yet been published.

### Everybody's Opinion.

[Correspondence on all subjects is invited, but we cannot in any way be responsible for the opinions expressed by our correspondents. No communication can be entertained if the name and address of the correspondent are not given as a guarantee of good faith, but not necessarily for publication. All correspondents should write on one side of the paper only.]

### AMERICA AND ENGLAND.

"COSMOPOLITAN" writes: In answer to "American Nurse" and the sweeping assertions she makes in your last issue of our doctors and nursing homes, and as an 18 years' resident of the country she hails from, allow me to publicly repudiate the gross libels on a body of the gentlest and bravest men existing—our English doctors. "American Nurse's" experience must be cast amongst the Western States where any kind of quack, provided he has engaging manners and plenty of "soft soap" with hysterical women and disloyal nurses, may hang out a "shingle" and proclaim himself "Doctor" without let or hindrance. No, "American Nurse," our doctors are second to none as gentlemen, and our trained nurses are proud to be the helpers and servants (if you will) of the brave men always ready to alleviate misery and pain. Our nurses do not look for flattery for doing their duty, and our doctors, while treating a good nurse with courtesy, remember that "familiarity breeds contempt." The result is a loyalty towards our English doctors which "American Nurse" would do well to copy.

D. Z. BEAUMONT, 104 Church Road, Upper Norwood, writes: It seems to me very surprising that "An American Nurse" can so misrepresent the state of nursing in England. Can it be that she is both incompetent and disappointed, for I venture to think that she is very much in the wrong when she says that the nursing profession is about as bad as it can be just now in England. It is as absurd as untrue to say that "specialists" dress their housemaids in nurses' uniforms and charge three guineas a week for their services; nor is it any more correct to say that those admirable institutions "nursing homes," palm housemaids and lady-helps on the public who, by their vulgarity and want of training bring degradation on the nursing profession. I have seen for some years a great deal of lady nurses, and I have found them uniformly to be very ladylike and attentive, though it is needless to say that in the nursing, as in every other profession, there are of course many exceptions. I cannot admit that there is any higher standard of duty among nurses in America than in England, and I have not found that the doctor can be honestly called "Lord Pills," nor that the nurses are frequently treated as servants.

"AN ENGLISH NURSE" writes: The letter from "An American Nurse" is evidently from one who came over to England on speculation expecting to get good work. The Editor of THE HOSPITAL is always advising nurses not to go abroad without being engaged first, and if only nurses would believe that it is no earthly use going to another country unless they are definitely engaged beforehand, they would be saved much disappointment; also they must know something of the manners and customs of the people they go amongst, and the language, and realise that they only go abroad to nurse those of their own nationality, for foreigners do not (as some think) want to be nursed by Englishwomen any more than English people like to be nursed by nurses of other countries. Our American cousin probably came over with the best of American certificates to find that their value was not appreciated and she was regarded with suspicion; then we should gather from her letter she tried calling on doctors, hoping to get cases; there again she would be disappointed, for English doctors prefer their own nurses, trained in their own hospitals, under their own eyes. Finally, the American nurse would appear to have landed in a third-rate nursing home, through whose windows she judged the English medical and nursing professions. It is a pity that she should have allowed her bitter feelings to run away with her and tell us that English doctors dress up their servants in hospital uniform to nurse their sick patients. We all know this to be so utterly untrue that it hardly needs refuting; no English surgeon or physician would risk the life of his patients or his own reputation by so doing, when he can have as many trained nurses as he wants. We feel sorry for "An American Nurse," for we should no doubt feel as bitterly disappointed as she does did we try to get work for ourselves in New York, and she has evidently not had the chance to see good English nursing or she would know that English medical men and women and their hospital nurses work together as comrades and both try to minister their best to their patients whether they are ill bred or otherwise.

### ARE NURSES LOYAL?

"SISTER" writes: I was most interested reading the letter headed "Are Nurses Loyal?" and am very much in sympathy with all the writer's views. I have done private nursing myself and know only too well what a monotonous and thankless existence it is. Nobody can possibly dispute the fact that unfortunately ofttimes it is taken up by persons absolutely unsuitable for it, and those who care more to study their own enjoyment than the welfare of their patients; still it seems hard that the whole profession should be condemned and stated to be composed of bad and immoral women on account of the few who have most apparently missed their vocation. If nurses themselves would strive to be more loyal to each other this perhaps might in a measure be prevented—for surely in every class of women there are some who have shown themselves unworthy of their calling and fallen short of what was expected of them. If we constantly see and hear of a hospital-trained woman sneering at one with only infirmary training, to my mind it only shows that the former's training is still incomplete. I hold a three years' general hospital certificate, so to me this does not apply, but I am now filling the post of ward sister at one of the large infirmaries outside London, and cannot say enough or speak too highly in favour of the nurses. We are not well staffed as the Poor Law only allows us two nurses to 28 patients, but the thoroughness, kindness, and extreme self-sacrifice shown on the part of these nurses would often put hundreds of hospital-trained women to shame. Training alone I consider goes for little if the personal qualities so essential to a good nurse are not present.

### ARMY SISTERS AND ORDERLIES.

"AUBREY" writes: May I as a male nurse trespass upon your valuable space to enter a protest against the letter of "J. M." in your issue of the 18th inst., and to endeavour to refute at least one of his statements? I have read his extraordinary communication several times and I confess that the more I read it the more obscure it becomes, and although I am not in a position to judge of the merits of his particular case, it certainly reads like the vulgar abuse of one who is lacking in some of those qualities which go to the making of a good nurse, and suggests much of that inferiority which engenders hatred. It is lamentable that the state of things which "J. M." describes should exist in the Royal Army Medical Corps, and I deprecate the airing of this grievance in the columns of your journal as such a course is likely to injure the profession of which he appears to be so distinguished a member. Surely a more libellous letter was never penned to disparage the Army sisters, and although they stand in need of no defence from me, my whole nature revolted at the unjust and supercilious tone of "J. M.'s" letter, and I felt that I could not let the letter pass without making a protest and quoting my humble experience. I have met several army nurses, both sisters and staff nurses (the latter class "J. M." seems to be unaware of) and they in no way deserved any disparaging criticism. If your correspondent has the misfortune to work with a sister who is not all she should be, may I suggest that if a disagreement is inevitable there is no reason why it should engender rudeness or bitterness, and if the qualities of chivalry, patience, and gentleness were more cultivated in the Royal Army Medical Corps it would do much to smooth away the difficulties which now exist. There is one part of your correspondent's letter I take special exception to—namely, in speaking of "mixed nursing" as the cause of much friction in the Royal Army Medical Corps, when he says: "The wonder is that it has gone on so long without even more fury than now threatens the very extinction of one or the other." Now I emphatically deny that mixed nursing is such an evil, and my humble observation and experience tend to prove that male and female nurses can work in the same ward and engage in private nursing without any friction whatever. *A propos* of this I might mention that there are four nurses engaged in nursing my present patient, two of whom are female and there has not been the slightest unpleasantness. My previous patient, whom I nursed single handed for two years, had a near relative who was a nursing sister at a large hospital, and this lady used periodicaly to assist with the nursing, and I can truthfully say that there was no contretemps whatever in all that time. And mine is not a solitary case, as for many years there have been working side by side in the wards of the National Hospital, London, male and female nurses, in perfect discipline and harmony. Moreover, far from being undesirable, "mixed nursing" properly carried out works frictionless and makes for efficiency because the *esprit de corps* of each sex would prompt them to do their very best in the presence of the other, and the chivalry of the man prevents any unseemly bickerings to the material advantage of the patients, for where such friction as "J. M." describes exists, the patients cannot get their proper attention. In conclusion, when your correspondent talks of "the feud which has existed since 1884, and that while it could never be discovered that a Royal Army Medical Corps man had killed a sister frequent court martials have taken place as a result of the sisters' reports." He has provided some melodramatic details for the benefit of your readers.

### Appointments.

Bradford Children's Hospital.—Miss Nora Woodhouse has been appointed matron. She was trained at Sunderland Infirmary, where she has since been sister. She has also been sister at the Children's Infirmary, Liverpool, and sister at the Children's Hospital, Manchester.

Buchanan Hospital, St. Leonards.—Miss Esther Hodges has been appointed matron. She was trained at the Charing Cross Hospital, where she was eight years, four years of which she was sister of medical and surgical wards and theatre sister. She was also two years as nurse at the Chelsea Hospital for Women.

Burton-on-Trent General Infirmary.—Miss Lydia Louise Rose has been appointed sister. She was trained at the Walsall and District Hospital, and has since been sister at the Guest Hospital, Dudley, sister at Wolverhampton General Hospital, and sister at Walsall and District Hospital.

City Hospital, Fazakerley, Liverpool.—Miss Winifred Redding has been appointed charge nurse. She was trained at St. Mary (Islington) Infirmary, where she has since been staff nurse. She was also nurse at the Gore Farm Fever Hospital, Hampstead.

Cottage Hospital, Swaffham.—Miss Mabel S. Kennedy has been appointed matron. She was trained at the County Hospital, York, and has since been ward sister, home sister, and night sister at the same institution and sister-in-charge of the Nurses' Home, Beccles, and district nurse at Thetford, Norfolk.

Hereford Memorial Hospital and Sanatorium, Alcaster.—Miss Ada J. Farr has been appointed matron. She was trained at the Monsall Hospital, Manchester, and has since been occupied in private nursing and as matron of the Wood Green Isolation Hospital, Finsbury Park.

Infectous Diseases Hospital, Ashton-in-Makerfield, Lancashire.—Miss C. G. Mulcahy has been appointed charge nurse. She was trained at St. George's Infirmary, Fulham Road, and was afterwards charge nurse there. Since then she has been charge nurse at the Fullerton Accident Hospital, Yorkshire, and been occupied in private nursing.

Much Wenlock Hospital.—Miss Mary Smith has been appointed sister. She was trained at the Royal Infirmary, Manchester, where she has since been staff nurse.

Union Infirmary, Wellington, Somersetshire.—Miss Lillie Emma Baker has been appointed charge nurse. She was trained at Eastville Workhouse Infirmary, Bristol, where she has since been staff nurse. She has also been staff nurse at Bath Medical and Surgical Home.

### The Nurses' Bookshelf.

Handbook for Midwives and Maternity Nurses. By Comyns Berkeley, M.B., etc. London: Cassell and Company, Ltd., 1906. Pages 283; illustrations 58. Price 5s. Size, $6\frac{3}{4}$ in. by $4\frac{1}{4}$ in.

This is a neatly got-up little book, printed clearly and well illustrated. We like the arrangement of the work. It is divided into six parts. The first part deals with the anatomy of the pelvis of the female generative organs, of the placenta and the fœtal membranes and of the fœtus. Part II. deals with the physiology and the pathology of pregnancy. Next we come to labour, treated of in the same way, and so on as regards Parts IV. and V., in which the puerperium and the infant respectively are considered, a few useful remarks being added on the management of children born prematurely. The last part of the book (Part VI.) is concerned with the important subjects of the elements of house sanitation and asepsis and antisepsis in midwifery, and this is perhaps the most important part, the different antiseptics in common use being considered and the strength of the solutions required for various purposes. Turning to details, on page 6 the interspinous and intercristal diameters are given, but the importance of a definite relation of the one to the other is not insisted upon. We have always regarded the oblique diameters of the cavity of the pelvis as measuring $4\frac{1}{2}$ inches, and not 5 inches as stated in the text. We can find no mention made of the deciduæ, of the physiology of conception, or of the fœtal circulation. Extra-uterine gestation, too, is not referred to. On page 97, in describing the result of flexion, it is stated that "the occipito-frontal diameter, which measures $4\frac{1}{2}$ inches, becomes substituted for the sub-occipito-bregmatic, which measures $3\frac{3}{4}$ inches." Surely this should be *vice versâ*. On page 268, the fifth line from the bottom, we notice a misprint. Many parts of the book are admirable in their simplicity and conciseness, but the book has been too much condensed, and thereby its usefulness is diminished.

### A Book and its Story.

### A SPINSTER'S PROGRESS.*

Wilhelmina Castel, whose adventures and vicissitudes are related by Mr. Barry Paine with a bold disregard to ordinary happenings and a lively perception of the humorous effects that result, is in every sense a heroine. Self-reliant, practical, and not daunted by obstructions which beset the path of beauty unattended, she starts out, on the death of an impecunious father, to conquer circumstances and make a name for herself. Two possessions are hers: an inherited spirit of adventure and a sense of humour. These combined lead into, and out of, some unusual situations. When her story opens she has still a father. Her mother had died when Wilhelmina was a child. At first her father directed her. Then, as soon as it was possible, Wilhelmina directed her father. This apparently is the position of affairs when the book begins. On the opening page we read: "It is quite possible to love a person whom one does not respect, of whom one even disapproves. I loved my father, but I certainly did not respect him. He did not even respect himself. When he married my mother, much against the wishes of his family, my grandfather bought him an annuity of two hundred a year, and desired to have nothing more to do with him." In moments of financial difficulty, which occurred frequently, Mr. Castel sought immunity from present anxieties by making imaginary provisions for the future. He drew up innumerable wills. The last one made began: "I, Bernard Castel, being of sound mind and at peace with God and man, do hereby give and bequeath all my real and personal estate, of whatsoever kind, to my only beloved daughter, Wilhelmina." Then follows directions for the disposal of an imaginary estate. "At that time we were as usual skating on the very edge of bankruptcy. . . . I suppose he really loved me. He often told me when a financial crisis was at its worst that I was all he had in the world. But he never insured his life, and never made any provision for me after his death. . . . The trouble of it was that my father could never let his income alone. Every quarter-day brought some new scheme, generally of a wildly speculative character. At first my father confided these schemes to me, but I am quite practical and hated them, and told him so. Then he kept his schemes to himself, merely observing when the bottom had dropped out of them, 'Wilhelmina, I fear I have made a fool of myself again. . . .'" But Mr. Castel had a source of income, small certainly, but one which might have been increased had he spent as much time in its pursuit as in less remunerative schemes. He wrote short stories, "of the most extreme sentimentality and of the most aggressively moral character. He and I have screamed over them many a time. . . . He did not take his literary work seriously at all, and it used to be my chief amusement to get him to read out his own stories, with his own parenthetical comments." At the time of Mr. Castel's death he and Wilhelmina were living in a village of that name—Castel-on-the-Weld—and chosen on this account as a place of residence, solely. He had come on the name in Bradshaw, and thought it 'would be nice and hereditary' to be Bernard Castel, Esquire, of Castel-on-Weld. I am not aware that any of his ancestors had ever lived within a hundred miles of the place. . . . He was popular, as most extravagant men with a sense of humour are, but his humour had a blind point. He could never see that any of his wild-cat business was utterly ridiculous, or understand why sometimes in the middle of our deepest distress I could not help laughing at him. . . . During his last illness several people to whom he owed money for a long time sent him presents. I thought it was rather touching." Wilhelmina's father dies, and she is left to face the world alone. Her grandfather has sent her a cheque for two hundred pounds, enclosed in a letter informing her, "in a courteously acidulated way," that he had no wish to see her and that she had nothing to expect from him. Wilhelmina pays her father's debts and then reflects on the future. "I had thought it all out. . . . I did not want to be a companion. . . . I had clearly made up my mind that there was no sort of work in the world that I should be ashamed to do if I could do it, but that I could not take anything which could not possibly lead to anything. . . . I had vague ideas that I should like to get into some kind of business, and by cleverness and practicality and temperance, and early rising, and the rest of the bag-of-tricks, worm my way slowly upwards until I was manageress and indispensable. All the time I should be saving money, and should then be ready to start myself. . . . I think I was well educated, though rather in a general and erratic way. It is possible with my knowledge of French, music, and literature I might become a governess." But, anyhow, before taking steps in either direction, Wilhelmina decides naturally that she must get out of Castel-on-the-Weld, because things did not happen there. The doctor and the rector had both come forward with friendly advice. The former refused at first to take any fee for his medical attendance on Mr. Castel; he said that medical etiquette did not allow of his making any charge to an orphan girl, and that if he took my money he would be hounded out of the profession, and quite properly. "But . . . I made him take the money. . . . He had given us any amount of his time and had charged nothing for it. He was not a rich man either."

Marriage had also entered to Wilhelmina's mind, but only in the abstract. She was eighteen and the world was before her. "I had also thought of marriage. Even if I had not thought of it, the fact that the doctor had proposed to me twice would have reminded me of it. I was pretty enough, and though the idea of falling in love had not occurred to me, I thought that I might marry rather well one day. This was an additional reason for leaving Castel-on-the-Weld." So Wilhelmina departs for London.

"I was going to play a lone hand, and the honest truth is that I rather enjoyed the prospect. I was going to London, the place where things happen, to do what I wanted in the way I wanted. Perhaps I should starve and perhaps I should have fun. . . . I had no clear idea what I was going to do, but I had got new clothes, no debts, and about seventy-five pounds in cash. . . . So one morning I got into a third-class railway carriage, and an old woman talked to me at length about her somewhat unseemly physical infirmities. I hope I was sympathetic, but my mind was already in London, looking out for the possibility of adventure." Wilhelmina does not have to wait long for an excitement. Upon her arrival at Charing Cross she was struck by a number of girls waiting on the platform. They were dressed in the same style as herself. In height, colouring, and general appearance they bore a striking resemblance to herself. They were evidently waiting for someone. What happens in this first adventure of Wilhelmina's we must not say. But in this and others, equally unusual, we hope our readers will follow this original heroine, as she leads them on in her book from one thrilling experience to another. The story comes to a happy, but not a "lone" conclusion.

* "Wilhelmina in London." By Barry Paine. (John Long. 6s.)

### Notes and Queries.

**REGULATIONS.**

**The Editor is always willing to answer in this column, without any fee, all reasonable questions, as soon as possible.**

**But the following rules must be carefully observed.**

1. **Every communication must be accompanied by the name and address of the writer.**
2. **The question must always bear upon nursing, directly or indirectly.**

**If an answer is required by letter a fee of half-a-crown must be enclosed with the note containing the inquiry.**

*India.*

(247) How can I get out to India, passage free, as my people live there?—*Birmingham.*

You must advertise in the daily and nursing papers that you will give your services in exchange for your passage; but it may be some time before you can make a satisfactory arrangement.

*St. John's Ambulance.*

(248) Are there any St. John's Ambulance classes before 10 A.M. and after 7 P.M. I cannot attend between these hours? *P. V.*

Write to the St. John's Ambulance Association, Clerkenwell, E.C.

*Mental.*

(249) Can you tell me of a Home where a young girl suffering from spinal curvature and mentally deficient can be received? She is able to do light duties, and can pay a little.—*Sister.*

If not a suitable case for one of the county asylums, write and ask for advice from the National Association for Promoting the Welfare of the Feeble-minded, 53 Victoria Street, London, S.W., enclosing stamped envelope.

*Sick Cookery.*

(250) How can I get lessons in invalid cookery quickly?—*Spes.*

Possibly one of the hospitals, such as the London, might allow you to join their classes. "How to Feed the Invalid" (London, the Scientific Press, 28 Southampton Street, Strand, W.C.) is a very good book.

*Hospital Training.*

(251) Where can a lady secure a short training? She is above the usual age.—*Betsie.*

If she is under forty years of age she would be received at several hospitals as a paying probationer for three months or more. Try St. Bartholomew's, St. Mary's, Paddington, or University College Hospital.

*Canadian Certificates.*

(252) If a nurse trained in Canada would her certificate hold good in England or vice versa?—*Dublin.*

A certificate from an important hospital in either country would be recognised.

*Ruptured Arteries.*

(253) A patient who has ruptured arteries of the ankle has now a swollen and flabby leg. Is the flabbiness water, and is an elastic stocking preferable to a bandage?—*Nurse.*

We are surprised that a trained nurse should not know that the case she refers to should be at once placed in the hands of a medical man.

*Nursing Homes.*

(254) Can you tell me of a nursing home in Paris?

We are unable to recommend any, but possibly the matron of the Hertford British Hospital, 72 Rue des Villiers-Levallois-Perret, might help you.

*Nursing in Switzerland.*

(255) Is a trained nurse likely to obtain work in Switzerland in the summer?

Yes, if she make the acquaintance of both English and Swiss doctors in the town where she wishes to work.

**Handbooks for Nurses.**

Post Free.

"How to Become a Nurse: How and Where to Train." 2s. 4d.
"Nursing: its Theory and Practice." (Lewis.) ... 3s. 6d.
"Nurses' Pronouncing Dictionary of Medical Terms." 2s. 6d.
"Complete Handbook of Midwifery." (Watson.) ... 6s. 4d.
"Preparation for Operation in Private Houses." ... 0s. 6d.

Of all booksellers or of The Scientific Press, Limited, 28 & 29 Southampton Street, Strand, London, W.C.

### For Reading to the Sick.

### SPIRITUAL COMPANIONSHIP.

A rustling as of wings in flight,
An upward gleam of lessening white,
So passed the vision, sound and sight.

But round me, like a silver bell
Rung down the listening sky to tell
Of holy help, a sweet voice fell:

"Still hope and trust," it sang; "the rod
Must fall, the wine-press must be trod,
But all is possible with God!"

*J. G. Whittier.*

A true acceptance of the whole Bible idea of ever-present spiritual life would not set us watching . . . for the sight of angels, but it would give us the strength which comes to every work and suffering from the knowledge that this universe is larger than it seems, and that it is all peopled with spiritual existences who are God's ministers to enlighten and to feed our life. . . . The mother may not discern an angel bending over the bed on which her child is laid, but still she may know that there are other watchers by its bed beside herself, spirits whom God has sent to see that none of His little ones take any harm. And I cannot but think that it will change our whole idea of death. Surrounded by this spiritual life, and yet seeing it only here and there through broken gaps of this enveloping mortality, what will it be for us to die? Only to cast this mortality away and stand face to face with the realities that have been close to us all the while. All spiritual companionship, all unknown spiritual protection that has been blessing us in the darkness opened suddenly into the light, so that we see it all, and enter on the new life that begins with death.—*Bishop Phillips Brooks.*

We are apt to feel as if nothing we could do on earth bears a relation to what the good are doing in a higher world; but it is not so. Heaven and earth are not so far apart. Every disinterested act, every sacrifice to duty, every exertion for the good of "one of the least of Christ's brethren," every new insight into God's works, every new impulse given to the love of truth and goodness, associates us with the departed, brings us nearer to them, and is as truly heavenly as if we were acting, not on earth, but in Heaven. The spiritual tie between us and the departed is not felt as it should be. Our union with them daily grows stronger, if we daily make progress in what they are growing in.—*Channing.*

The angels of the Lord are ever found
Encamped about the soul that looks to Him:
They are an inner lamp when all is dim
Without . . . . . . . . . . . . . . .
Even as a myriad sunbeams hour by hour
Melt to make rich one little summer flower;
Or as a myriad souls of flowers fleet
Away to make a single summer sweet—
So many spirits make one smile of God
That feeds your life transfiguring from its clod.

*Gerald Massey.*

## Figures and Tables

**Fig. 19. f1:**
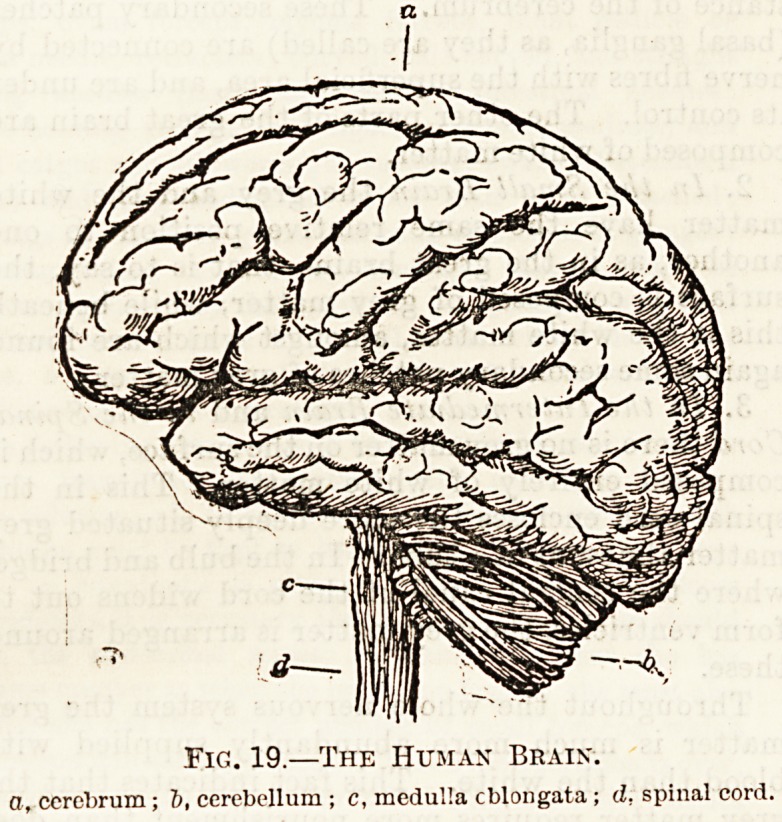


**Fig. 20. f2:**
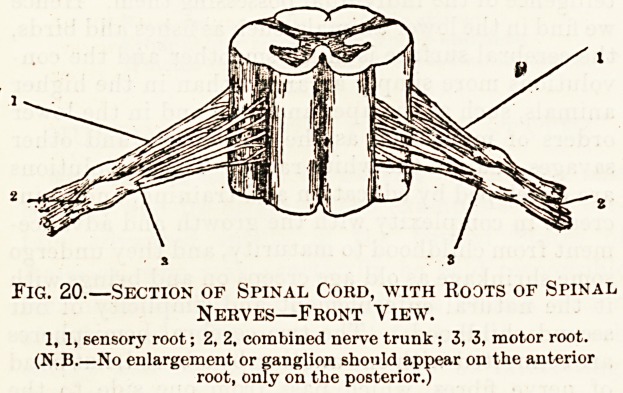


**Figure f3:**